# CMT2N-causing aminoacylation domain mutants enable Nrp1 interaction with AlaRS

**DOI:** 10.1073/pnas.2012898118

**Published:** 2021-03-22

**Authors:** Litao Sun, Na Wei, Bernhard Kuhle, David Blocquel, Scott Novick, Zaneta Matuszek, Huihao Zhou, Weiwei He, Jingjing Zhang, Thomas Weber, Rita Horvath, Philippe Latour, Tao Pan, Paul Schimmel, Patrick R. Griffin, Xiang-Lei Yang

**Affiliations:** ^a^Department of Molecular Medicine, The Scripps Research Institute, La Jolla, CA 92037;; ^b^School of Public Health (Shenzhen), Sun Yat-sen University, 510006 Guangzhou, China;; ^c^Department of Molecular Medicine, The Scripps Research Institute, Jupiter, FL 33458;; ^d^Department of Biochemistry and Molecular Biology, University of Chicago, Chicago, IL 60637;; ^e^School of Pharmaceutical Sciences, Sun Yat-sen University, Guangzhou 510006, China;; ^f^Shanghai Key Laboratory of New Drug Design, School of Pharmacy, East China University of Science and Technology, 200237 Shanghai, China;; ^g^Dynamic Biosensors GmbH, 82152 Martinsried, Germany;; ^h^Department of Clinical Neurosciences, University of Cambridge, Cambridge, CB2 0PY, United Kingdom;; ^i^Biology and Pathology Department, Hospices Civils, 68500 Lyon, France

**Keywords:** Charcot-Marie-Tooth disease, neuropilin 1, AlaRS

## Abstract

Through dominant mutations, aminoacyl-tRNA synthetases constitute the largest protein family linked to Charcot-Marie-Tooth disease (CMT). An example is CMT subtype 2N (CMT2N), caused by individual mutations spread out in AlaRS, including three in the aminoacylation domain, thereby suggesting a role for a tRNA-charging defect. However, here we found that two are aminoacylation defective but that the most widely distributed R329H is normal as a purified protein in vitro and in unfractionated patient cell samples. Remarkably, in contrast to wild-type (WT) AlaRS, all three mutant proteins gained the ability to interact with neuropilin 1 (Nrp1), the receptor previously linked to CMT pathogenesis in GlyRS. The aberrant AlaRS-Nrp1 interaction is further confirmed in patient samples carrying the R329H mutation. However, CMT2N mutations outside the aminoacylation domain do not induce the Nrp1 interaction. Detailed biochemical and biophysical investigations, including X-ray crystallography, small-angle X-ray scattering, hydrogen-deuterium exchange (HDX), switchSENSE hydrodynamic diameter determinations, and protease digestions reveal a mutation-induced structural loosening of the aminoacylation domain that correlates with the Nrp1 interaction. The b1b2 domains of Nrp1 are responsible for the interaction with R329H AlaRS. The results suggest Nrp1 is more broadly associated with CMT-associated members of the tRNA synthetase family. Moreover, we revealed a distinct structural loosening effect induced by a mutation in the editing domain and a lack of conformational impact with C-Ala domain mutations, indicating mutations in the same protein may cause neuropathy through different mechanisms. Our results show that, as with other CMT-associated tRNA synthetases, aminoacylation per se is not relevant to the pathology.

## Significance

Charcot-Marie-Tooth disease (CMT) is a devastating motor and sensory neuropathy with an estimated 100,000 afflicted individuals in the US. Unexpectedly, aminoacyl-tRNA synthetases are the largest disease-associated protein family. A natural explanation is that the disease is associated with weak translation or mistranslation (caused by editing defects). However, our results with six different disease-causing mutants in AlaRS ruled out defects in aminoacylation or editing as causal factors. Instead, specific mutant proteins gained a neuropilin 1 (Nrp1)-AlaRS interaction. Previously a gain of Nrp1 interaction with a different disease-causing tRNA synthetase was mechanistically linked to the pathology of CMT. Thus, our results raise the possibility that pathological engagement of Nrp1 is common to at least a subset of tRNA synthetase-associated cases of CMT.

Charcot-Marie-Tooth disease (CMT) is the most common inherited neurological disorder, affecting 1 in 2,500 people globally, with no treatment available beyond supportive care (1). Also known as hereditary motor and sensory neuropathy (HMSN), the disease predominantly affects peripheral nerves that control muscle movements and also carry sensory information to the brain, leading to muscle weakness and loss of sensation, especially in the hands and feet. Genetically speaking, CMT is a heterogenous group of diseases that have been linked to more than 100 protein-coding genes (2). Although for some genes their connection to peripheral nerve structure and/or function is obvious, this is not the case for many other genes. Prominently among them are the genes encoding aminoacyl-transfer RNA (tRNA) synthetases (aaRSs). So far, five aaRSs (i.e., glycyl [GlyRS or GARS1]-, tyrosyl [TyrRS or YARS1]-, histidyl [HisRS or HARS1]-, alanyl [AlaRS or AARS1]-, and tryptophanyl [TrpRS or WARS1]-tRNA synthetases) have been unequivocally linked to the disease, constituting the largest gene family implicated in CMT (2, 3).

aaRSs are evolutionarily conserved essential enzymes responsible for charging tRNA with their cognate amino acids to support ribosomal protein synthesis (4). In complex multicellular organisms, the functional landscape of aaRSs has been expanded with broad regulatory functions beyond their enzymatic activity (5). At least for three CMT-linked aaRSs, including GlyRS, TyrRS, and HisRS, a loss-of-function (enzymatic) mechanism has been excluded (6–12). In part this is due to the fact that CMT-linked mutations in aaRSs are dominant; patients always have both an affected and an unaffected gene allele, expressing the mutant aaRS along with the wild-type (WT) enzyme to support tRNA aminoacylation even when the mutant is defective (7). Interestingly, CMT-causing mutations in these three aaRSs all induce structural opening (6–8), which can render aberrant interactions outside the translation machinery that contribute critically to the disease pathology (6, 9, 13, 14). In particular, the aberrant interaction made by secreted CMT-mutant GlyRS with transmembrane receptor neuropilin 1 (Nrp1) inhibits VEGFA from binding and transmitting a neurotrophic signal through the receptor (9).

AlaRS is one of the aaRSs firmly linked to CMT, designated as subtype 2N (CMT2N), also known as AD-CMTax-AlaRS to denote the autosomal dominant inheritance and predominant axonal phenotypes (3). In contrast to other CMT-linked aaRSs, which are dimers, human AlaRS catalyzes the tRNA aminoacylation reaction as a monomer (15). This unique feature of AlaRS is particularly interesting because it has been shown for these dimeric aaRSs (i.e., GlyRS, TyrRS, and HisRS) that CMT mutation-induced structural opening is mainly localized at or near the dimerization interface (6–8). Would CMT-causing mutations in AlaRS also induce structural opening? If so, where would the opening site be?

**Fig. 1.**
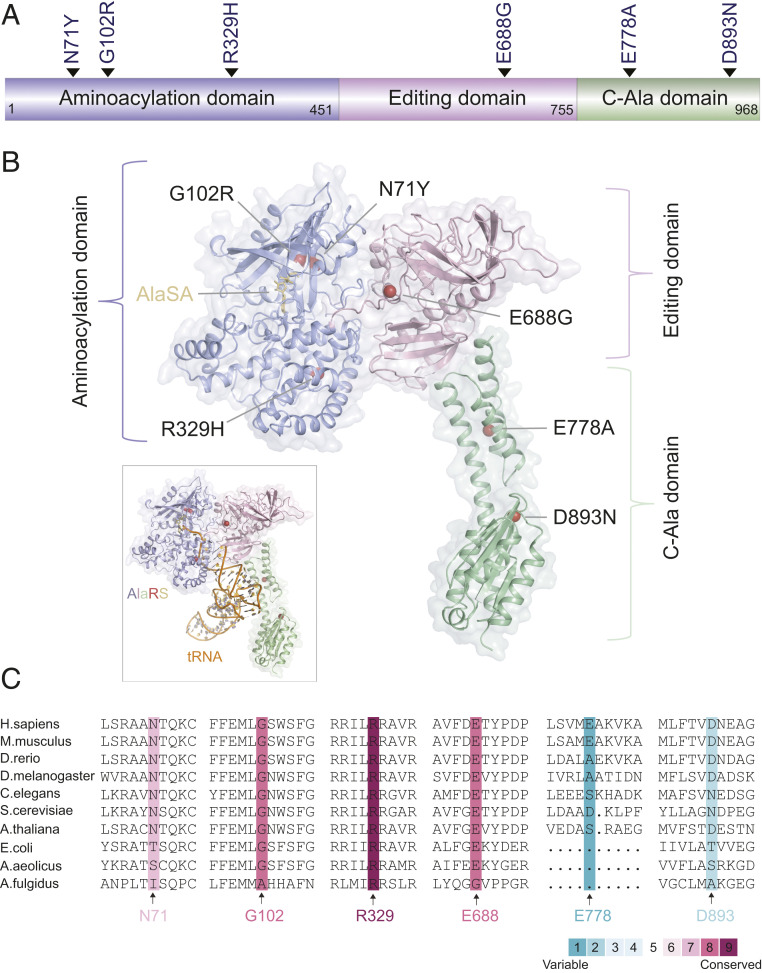
Distribution of CMT-causing mutations on AlaRS. (*A*) Six CMT2N-associated dominant mutations distributed in all three domains of cytosolic human AlaRS. (*B*) Assembled structure model of human AlaRS monomer. The model was first generated by SWISS-MODEL (45). The aminoacylation/catalytic domain (in complex with AlaSA, a Ala-AMP analog) and the C-Ala domain were subsequently replaced with their crystal structure (PDB 5KNN and PDB 5T5S, respectively) followed by manual adjustment. CMT mutation sites are indicated with red balls. *Inset* shows the structure model of human AlaRS and tRNA^Ala^ complex. The structure model of human AlaRS was aligned to *Archaeoglobus* *fulgidus* AlaRS in complex with tRNA^Ala^ (PDB 3WQY) according to the aminoacylation domain, and *A. fulgidus* tRNA^Ala^ sequence was replaced by human tRNA^Ala^. (*C*) Conservation analysis of AlaRS CMT mutation sites across eukaryotes, bacteria, and archaea.

AlaRS is also unique among CMT-linked aaRSs in that it contains an editing domain, located in between the aminoacylation domain at the N terminus and the C-Ala domain at the C terminus (Fig. 1*A*). The aminoacylation domain of AlaRS is necessary and sufficient in charging tRNA^Ala^ with its cognate amino acid alanine. It not only contains the catalytic active site but also key residues responsible for recognizing the major identity element of tRNA^Ala^, which is a G3:U70 wobble base pair in the acceptor stem (16). However, the AlaRS active site is not sufficient in selecting out the cognate amino acid due to high similarity of alanine with other amino acids, such as serine and glycine (17). Therefore, an editing domain has been incorporated into AlaRS to correct the mistake if it happens. The importance of the hydrolytic editing function of AlaRS has been extensively demonstrated, as even mild editing defects will cause severe diseases (18, 19). The function of the C-Ala domain in human AlaRS is not known. In prokaryotic AlaRS, C-Ala helps with tRNA binding, therefore enhancing enzymatic activity; however, this role is lost in humans (15).

CMT-causing mutations have been identified from all three domains of AlaRS (20–26). To broadly study the impact of CMT mutations on the structure and function of AlaRS, we included mutations from each domain, including three in the aminoacylation domain (N71Y, G102R, and R329H), one in the editing domain (E688G), and two in the C-Ala domain (E778A and D893N) (Fig. 1 *A* and *B*). Among them, the R329H mutation in the aminoacylation domain has been recurrently identified in eight unrelated families from four different countries, indicating ultrastrong disease-causing capacity of this mutation. We found that only N71Y and G102R, but not R329H in the aminoacylation domain, and no mutation from the editing and C-Ala domains, disrupt the catalytic activity. Consistently, no tRNA aminoacylation defect was detected in patient samples carrying the R329H mutation, confirming loss of function (enzymatic) is not the cause of CMT2N. None of the mutations, including E688G in the editing domain, affects the proofreading activity of AlaRS, suggesting CMT2N is not linked to a defect in editing function either. Mutations in the aminoacylation and editing domains induce a localized structural opening effect within each domain, whereas mutations in the C-Ala domain have no conformational impact. Interestingly, regardless of the differential impact on enzymatic activity, all mutations in the aminoacylation domain render an aberrant, gain-of-function interaction with Nrp1. The aberrant AlaRS-Nrp1 interaction is further confirmed in patient samples carrying the R329H mutation. This suggests that the gain-of-function impact of a mutation is a separate property from the enzymatic function. Moreover, this study provided evidence to indicate that even a nonenzymatic gain-of-function effect can be a shared disease mechanism among different tRNA synthetases.

## Results

### CMT2N Mutations Do Not Affect the Monomeric State of AlaRS or Reduce Protein Stability.

**Fig. 2.**
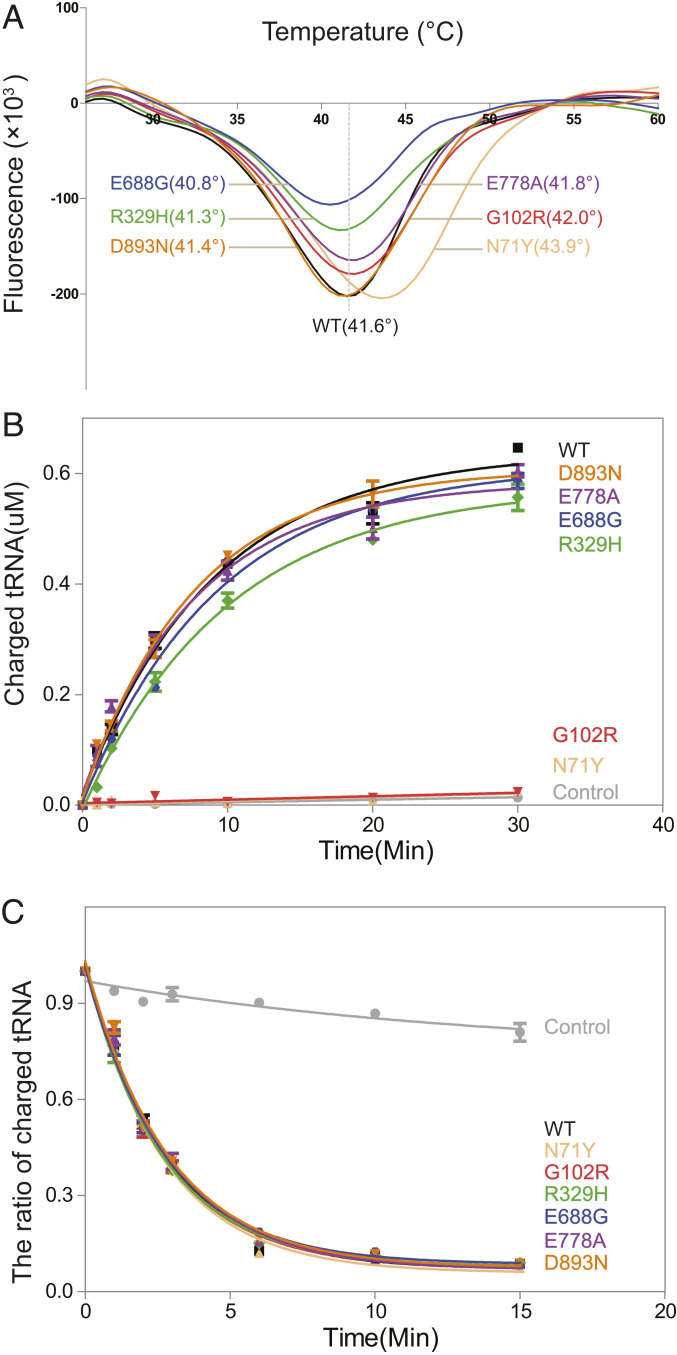
Enzymatic analyses of AlaRS and its CMT mutants. (*A*) Melting temperature (Tm) of AlaRS proteins determined by thermal shift assay. (*B*) Enzymatic activity of AlaRS proteins measured by in vitro aminoacylation assay with purified enzymes and in vitro transcribed tRNA^Ala^ as substrate. The reaction was negatively controlled with tRNA alone (no enzyme). Data are presented as mean ± SD (*n* = 2). (*C*) Editing activity of AlaRS proteins measured by deacylation of [^3^H] Ser-tRNA^Ala^. The reaction was negatively controlled with [^3^H] Ser-tRNA^Ala^ alone (no enzyme). Data are presented as mean ± SD (*n* = 2).

We first examined whether CMT mutations affect the monomeric state of AlaRS. Recombinant His-tagged AlaRS WT and six CMT mutation proteins were overexpressed in *Escherichia coli* and purified from the soluble fraction by nickel-nitrilotriacetic acid (Ni-NTA) column and ion-exchanging chromatography. All proteins displayed similar yields, suggesting that CMT mutations do not affect AlaRS stability. The gel filtration chromatography analysis confirmed that human AlaRS mainly exists as a monomer and that CMT mutations do not change the monomeric state of AlaRS (*SI Appendix*, Fig. S1). We also performed fluorescence-based thermal shift assays (TSAs). The melting temperature (T_m_) of all mutants is within 1 °C (plus or minus) from that of the WT AlaRS, except for N71Y, which has an increase of 2.3 °C in T_m_, indicating higher stability (Fig. 2*A*). Therefore, CMT mutations do not reduce protein stability of AlaRS.

### Majority of CMT2N Mutations Including R329H Do Not Impact tRNA Aminoacylation.

The AlaRS-catalyzed aminoacylation reaction occurs in two steps. In the first step, alanine is activated by adenosine triphosphate (ATP) in the active site of AlaRS, forming an enzyme-bound alanyl-adenylate (Ala-AMP) intermediate. In the second step, the activated amino acid is transferred to the 3′ end of tRNA to yield the alanyl-tRNA product, released together with AMP from the enzyme. To investigate the impact of CMT mutations on tRNA charging, we performed an in vitro aminoacylation assay that examines the overall reaction efficiency with purified recombinant AlaRS proteins and in vitro transcribed tRNA^Ala^. While N71Y and G102R, both located near the active site of AlaRS (Fig. 1*B*), completely abolished the enzymatic activity, R329H from the aminoacylation domain (but away from the active site), and mutations from editing (E688G) and C-Ala (E778A and D893N) domains have minor impact on the activity, if any (Fig. 2*B*).

### CMT Patients Carrying R329H Mutation Do Not Have a Defect in tRNA Aminoacylation.

**Fig. 3.**
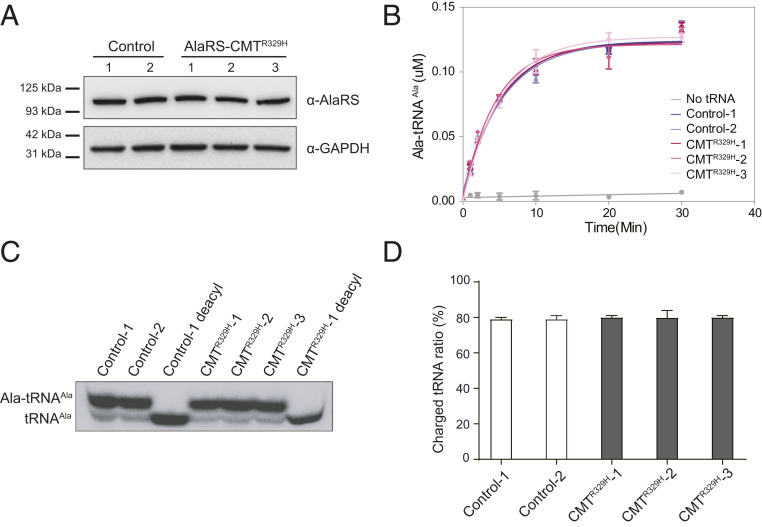
Cells from CMT patients carrying the AlaRS R329H mutation have no defect in tRNA^Ala^ aminoacylation. (*A*) Western blotting analysis indicated no change in AlaRS expression in peripheral blood mononuclear cell (PBMC)-derived lymphoblasts from three CMT2N patients with R329H mutation compared with two healthy people. (*B*) In vitro tRNA aminoacylation assay performed with cell lysates detected no activity defect in the CMT2N patients. In vitro transcribed tRNA^Ala^ was used as substrate. Data are presented as mean ± SD (*n* = 2). (*C*) Northern blotting analysis of total RNA extracted from PBMC-derived lymphoblast samples probed against cytosolic tRNA^Ala^. The RNA samples were loaded onto a denaturing gel to separate charged from uncharged tRNAs. Deacyl, deacylated samples included as controls. (*D*) Quantification of the ratio of charged-to-total tRNA^Ala^ presented as mean ± SD (*n* = 2 technical replicates per sample).

Because R329H was recurrently identified in CMT patients and was previously suggested to be a loss-of-function mutation (23), we examined lymphocytes derived from CMT2N patients carrying this mutation. Western blot analysis indicated no apparent change in AlaRS protein level in three different CMT patients compared with healthy individuals, confirming protein stability (Fig. 3*A*). Using the in vitro aminoacylation assay, we detected no enzymatic activity defect using lysates of CMT patient cells (Fig. 3*B*). Lastly, Northern blot analysis was performed to confirm the lack of deficiency in aminoacylation of endogenous tRNA^Ala^ in the patients (Fig. 3 *C* and *D*). Total RNAs were extracted from cells and analyzed under acidic conditions to prevent deacylation of charged tRNAs. About 80% of the endogenous tRNA^Ala^ was charged in cells from patients as well as from healthy individuals (Fig. 3*D*), an aminoacylation level that is consistent with expectation based on previous reports (27). Thus, we unequivocally concluded that CMT patients carrying the AlaRS R329H mutation do not have a defect in tRNA aminoacylation.

### Active Site of AlaRS^R329H^ Is Identical to that of WT AlaRS.

In parallel, we determined the crystal structure of the aminoacylation domain of AlaRS-R329H in complex with the nonlabile 5′-O-[*N*-(l-alanyl) sulfamoyl]adenosine (AlaSA) analog of the reaction intermediate Ala-AMP at 1.38-Å resolution (*SI Appendix*, Table S1). The aminoacylation domain of AlaRS by itself possesses robust activity in charging tRNA^Ala^ (28, 29). Our previous work solved the crystal structure of the aminoacylation domain of WT AlaRS (AlaRS^N455^) also in complex with AlaSA (30) (*SI Appendix*, Fig. S2*A*). However, using the same fragment for crystallization of the mutant protein, the resulting structure of AlaRS-R329H only contains the N-terminal 388 amino acids (aa), missing more than 60 aa from the C-terminal end (*SI Appendix*, Fig. S2*B*). This indicates that the C-terminal region of the aminoacylation domain is more flexible in AlaRS-R329H than in AlaRS-WT. Due to the increased flexibility, the C terminus might be too flexible to be resolved in the crystal structure or be cleaved by proteolysis during crystallization. Evidence in the crystal lattice interaction supports the latter explanation (*SI Appendix*, Fig. S2*C*). Indeed, AlaRS-R329H appears to be more susceptible to proteolysis than the WT protein (*SI Appendix*, Fig. S3). We further generated WT AlaRS^N388^ (by trypsin digestion of AlaRS^N455^) and determined its crystal structure at 1.28-Å resolution (*SI Appendix*, Fig. S2*D*) for comparison with that of R329H AlaRS^N388^. Superposition of WT AlaRS^N388^ and R329H AlaRS^N388^ structures shows a small rmsd of 0.36 Å for 348 Ca atoms and essentially identical active site and interactions with AlaSA (*SI Appendix*, Fig. S2 *E* and *F*). Consistently, the ATP/pyrophosphate (PPi) exchange assay that specifically examines the first step of the two-step aminoacylation reaction indicates no defect caused by the R329H mutation (*SI Appendix*, Fig. S4). Because the second step of the aminoacylation reaction is usually rate limiting (31, 32), defect in the first step may not be reflected by the overall aminoacylation efficiency. Nevertheless, we have confirmed that R329H mutation does not impact either step of the two-step aminoacylation reaction (Fig. 2*B* and *SI Appendix*, Fig. S4).

### CMT2N Mutations Do Not Impact the Proofreading Activity of AlaRS.

Next we examined the proofreading activity of AlaRS with premischarged serine-tRNA^Ala^ as the substrate. Although both serine and glycine can be misactivated by AlaRS, the Purkinje cell loss and ataxia phenotypes caused by a “sticky” mutation in the editing domain are more sensitive to serine than glycine mischarging (19). Therefore, we used serine-tRNA^Ala^ as the substrate for the deacylation assay. WT AlaRS as well as all six CMT mutants, including E688G located in the editing domain, efficiently hydrolyzed the mischarged tRNA (Fig. 2*C*). Therefore, CMT mutations do not impact the proofreading activity of AlaRS.

### Hydrogen-Deuterium Exchange Analysis Indicates Differential Effect of CMT Mutations on AlaRS Conformation.

The apparent susceptibility of AlaRS-R329H to proteolysis suggests increased conformational flexibility induced by the CMT mutation, reminiscent of what has been observed for other CMT-linked aaRSs (6–8). To pinpoint the site of conformational change in the context of the full-length protein and to compare different CMT mutations, we performed hydrogen-deuterium exchange mass spectrometry (HDX-MS) analysis for all six CMT mutants as well as the WT AlaRS as the reference. When the solvent of a protein is changed from H_2_O to D_2_O, amide hydrogens on the protein backbone undergo a hydrogen-to-deuterium exchange process. The rate of the exchange, or deuterium incorporation, represents solvent accessibility and conformation dynamics of each region in the protein (33). Mutation-induced conformational change in structure and dynamics can be inferred by comparing the mutant with the WT protein.

**Fig. 4.**
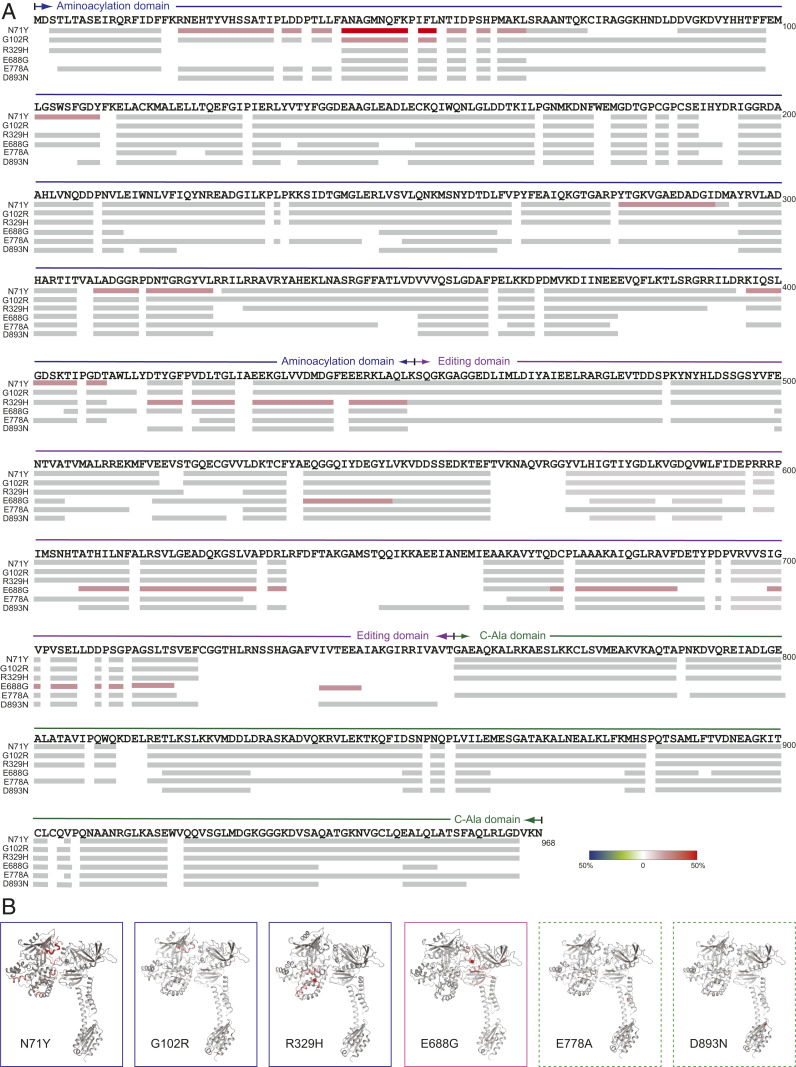
HDX analysis of human AlaRS CMT-causing mutations. (*A*) Changes in deuterium incorporation induced by each of the six CMT2N mutations mapped on the primary sequence of AlaRS. (*B*) Changes in deuterium incorporation mapped onto the structure model of AlaRS. Colored boxes indicate different domains where a CMT mutant is located. Blue: aminoacylation domain; purple: editing domain; green: C-Ala domain. Green boxes are dotted to indicate no change in deuterium incorporation.

Except for the two in the C-Ala domain, mutations located in the aminoacylation domain (N71, G102R, and R329H) and the editing domain (E688G) all showed an overall increase in deuteration incorporation, compared to WT AlaRS (Fig. 4*A*). The site(s) of conformational change, if any, is confined within the mutation-harboring domain. Particularly, mutations located within the aminoacylation domain only increase flexibility of the aminoacylation domain, and the editing domain mutation only increases flexibility of the editing domain, whereas no detectable conformational impact is induced by C-Ala domain mutations (Fig. 4*B*). Within the aminoacylation domain, G102R and R329H induce more localized conformational changes near the mutation site, whereas the impact of N71Y is more broadly distributed (Fig. 4*B*). Consistent with what we observed during crystallization and from the proteolysis analysis (*SI Appendix*, Fig. S3), R329H mutation causes increased flexibility at the C-terminal region of the aminoacylation domain (Fig. 4*A*).

### Small-Angle X-Ray Scattering Confirms Structural Opening Effect of Mutations in Aminoacylation and Editing Domains.

To confirm the differential effect of CMT mutations on AlaRS conformation, we performed small-angle X-ray scattering (SAXS) analysis on AlaRS proteins. For each protein sample, the shapes of the scattering curves are independent of the protein concentration (*SI Appendix*, Fig. S5*A*) and the corresponding Guinier plots show parallel and linear fits (*SI Appendix*, Fig. S5*B*), indicating the absence of significant aggregation during the measurements. The molecular masses extrapolated from the scattering curves are all around 110 kDa, consistent with a monomeric state for all AlaRS proteins, as indicated by gel filtration chromatography (*SI Appendix*, Fig. S1).

**Fig. 5.**
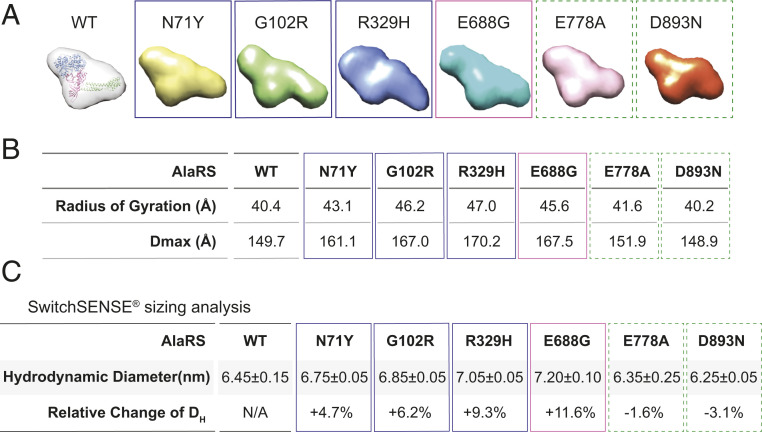
Conformational analysis of AlaRS and its CMT mutants. (*A*) SAXS-based ab initio envelope of AlaRS proteins. The structure model of AlaRS monomer was manually docked into the envelope of WT AlaRS. (*B*) Summary of SAXS parameters for the proteins tested. The radius of gyration value was determined from the Guinier plot using AutoRg and the maximum particle dimension (D_max_) was calculated using GNOM. (*C*) Summary of switchSENSE sizing data. Hydrodynamic diameter (D_H_) was presented as mean ± SD (*n* = 2). Colored boxes indicate different domains where a CMT mutant is located. Blue: aminoacylation domain; purple: editing domain; green (dotted): C-Ala domain.

The ab initio shape reconstruction analyses show a more elongated shape for mutants of aminoacylation and editing domains (i.e., N71Y, G102R, R329H, and E688G), compared with WT AlaRS and the C-Ala domain mutants (Fig. 5*A*). Consistently, the radius of gyration and maximum dimension (D_max_) derived from the scattering data are larger for all mutants except for the two in the C-Ala domain, compared to WT AlaRS (Fig. 5*B*). Interestingly, based on radius of gyration and D_max_ parameters, R329H induces the largest conformational change among all the aminoacylation domain mutations (Fig. 5*B*).

### Aminoacylation and Editing Domain Mutants Exhibit Larger Hydrodynamic Diameter.

We also used the switchSENSE technology to evaluate the hydrodynamic diameter of AlaRS proteins (34). This method tethers short double-strand DNA to a gold surface on one end and the other end was binding with fluorescence probe in a single-strand DNA and proteins of interest in the other strand. The motion of the DNA is triggered by alternating the voltage across the chip surface and is recorded in real time through the fluorescence probe attached to the DNA. Upon conjugation of a protein, the hydrodynamic friction of the DNA double strand is affected and subsequently the movement of these levers, which can be used to estimate the hydrodynamic diameter of the protein. Again, we found that all CMT mutants, except those of the C-Ala domain, have an increased size relative to WT AlaRS (Fig. 5*C*). Taken together, both SAXS analysis and hydrodynamic sizing data confirm that CMT mutations in aminoacylation and editing domains, and not the C-Ala domain, induce structural relaxation and opening of AlaRS.

### Only Aminoacylation Domain Mutations Induce Aberrant Interaction with Nrp1.

**Fig. 6.**
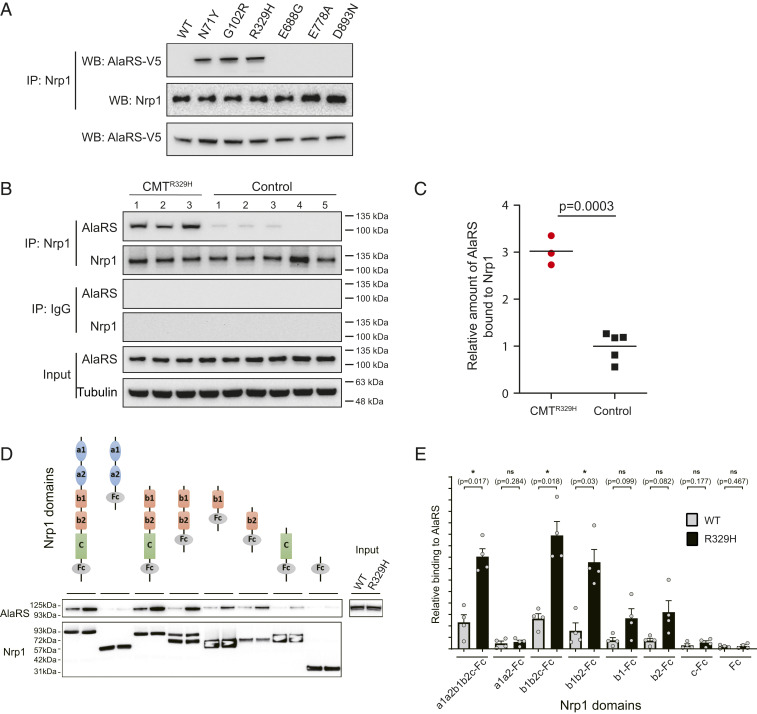
AlaRS-Nrp1 interaction. (*A*) Mutations in the aminoacylation domain, but not other domains, of AlaRS induce aberrant interaction with Nrp1. Nrp1-AlaRS interaction was detected by coimmunoprecipitation analysis using anti-Nrp1 antibody. AlaRS (WT or CMT2N mutants) was ectopically expressed in NSC34 cells with a V5 tagged and detected by anti-V5 antibody. (*B*) Coimmunoprecipitation analysis showing lymphocytes from CMT2N patients carrying the AlaRS R329H mutation (*n* = 3) give significantly stronger AlaRS-Nrp1 interaction than those from healthy individuals (*n* = 5). (*C*) Relative amounts of AlaRS bound to Nrp1 is presented by a scatterplot and quantified based on the amount of coimmunoprecipitated AlaRS relative to the amount of immunoprecipitated Nrp1 and the total AlaRS input. Unpaired Student’s *t* test was used for statistical analysis. (*D*) Domain mapping using pull-down experiments identifies the b1 and b2 domains of Nrp1 as primary binding site for CMT-mutant AlaRS. A representative Western blot from four independent experiments is shown. (*E*) Quantification of the pull-down experiments. Error bars represent the SE from the mean (SEM). *n* = 4. Statistical significance was determined by Student’s *t* test (paired, two tailed).

Previous studies indicated that the CMT mutation-induced structural opening in tRNA synthetases can render aberrant interactions outside the translation machinery that contribute critically to the disease pathology (6, 9, 13, 14). For example, CMT mutations in GlyRS induce an aberrant interaction of the synthetase with transmembrane receptor Nrp1 (9). Interestingly, while the WT AlaRS does not interact with Nrp1, all three CMT mutations in the aminoacylation domain can also induce an aberrant interaction with the receptor (Fig. 6*A*). However, not only the C-Ala mutations but also the editing domain mutation cannot induce Nrp1 interaction (Fig. 6*A*). Using patient-derived lymphocytes compared with those from healthy individuals, we confirmed the aberrant AlaRS-Nrp1 interaction in patients carrying the R329H mutation (Fig. 6 *B* and *C*).

The GlyRS^CMT^/Nrp1 interaction was mapped to the extracellular b1 domain of Nrp1 (9). To understand if the same domain in Nrp1 mediates the aberrant AlaRS^CMT^ intearaction, we tested various domain-deletion constructs of Nrp1 for binding to WT and R329H AlaRS (Fig. 6 *D* and *E*). Pull-downs of WT AlaRS and R239H with C-terminally Fc-tagged Nrp1 constructs showed that the extracellular a (a1 and a2) and c domains had no impact on AlaRS^CMT^ binding. By contrast, the Nrp1 b domains (b1 and b2) were sufficient to bind R329H AlaRS, with each domain contributing to the interaction. Since the two domains are adjacent to each other in sequence and tightly associated in the three-dimensional structure (35, 36), these results indicate that b1 and b2 together form a contiguous binding interface for mutant AlaRS. The b1 domain hereby constitutes the consensus binding site for CMT-associated mutant AlaRS and GlyRS.

## Discussion

Understanding how the disease mutations impact the structure and function of AlaRS, a unique member of the largest gene family causatively linked to CMT, is particularly interesting and important. AlaRS is the only CMT-associated tRNA synthetase that does not function as a dimer in tRNA aminoacylation and that has the need to incorporate a separate domain to perform hydrolytic editing function to correct the mistake when tRNA^Ala^ is mischarged with a noncognate amino acid. Both aspects are relevant to neurodegeneration. The dimer interface (mediated by the catalytic domain) has been shown over and over again, from GlyRS, TyrRS, to HisRS, to directly house and/or to be conformationally altered by many CMT-causing mutations, although the conformational change may not necessarily affect dimer formation (6–8). The editing function of AlaRS has been linked to neurodegeneration in a mouse model (19).

Typically, the tRNA aminoacylation function of an aaRS is supported by a catalytic domain harboring the active site and an anticodon binding domain recognizing the cognate tRNA. However, due to the lack of anticodon recognition of tRNA^Ala^, AlaRS has no anticodon binding domain. Instead, its aminoacylation domain not only contains the catalytic active site but also tRNA binding and recognition motifs. In this regard, the aminoacylation domain alone constitutes the catalytic core of AlaRS, whereas the catalytic core of the other CMT-associated aaRSs contains both catalytic and anticodon binding domains. Almost all confirmed CMT-causing mutations in other aaRSs are located within the catalytic core, whereas in AlaRS they are spread throughout the catalytic core, editing domain, and the C-Ala domain (*SI Appendix*, Fig. S6).

Some common schemes have arisen from past studies of CMT-associated aaRSs. First, despite their concentration in the catalytic core, they do not necessarily affect tRNA aminoacylation activity of the synthetase, as exemplified by GlyRS-E71G, TyrRS-E196K, and HisRS-D364Y, all of which have WT-like enzymatic activity (6, 12, 37, 38). Second, regardless of their impact on enzymatic activity, all CMT-causing mutations induce an open conformation in the mutant proteins relative to their respective WT counterpart. The open conformation is commonly featured by structural relaxation at the dimer interface that leads to an overall size expansion (6–8). Within each aaRS, the open conformation induced by various mutations are similar, albeit with different levels of dynamics and flexibility (6–8). This open conformation endows the mutant proteins with the ability to make aberrant interactions with other molecules. So far, we have identified at least four aberrant interaction partners of aaRS mutants that contribute to the pathogenesis of CMT. These include transmembrane receptor Nrp1, Trk receptors, and α-tubulin deacetylase HDAC6, which bind to GlyRS CMT mutants, and transcription repressor TRIM28 interacting with TyrRS CMT mutants (6, 9, 13, 14, 39).

In this study, we performed a structure–function study for AlaRS CMT mutants. Six different CMT mutations from all three domains of AlaRS were included in the study. Consistent with past studies, CMT mutations do not necessarily affect the tRNA aminoacylation activity of AlaRS. As expected, mutations in the editing domain and the C-Ala domain have no effect on charging (Fig. 2*B*). Notably, even among mutations located within the aminoacylation domain, the R329H mutation has little impact on enzymatic activity (Fig. 2*B*). The fact that R329H is recurrently identified and has the strongest association with CMT highlights once again the disconnection between enzymatic activity and CMT pathology. Importantly, the lack of enzymatic activity defect has been rigorously confirmed in patient samples (Fig. 3).

It is worth noting that four of the six mutations were previously studied for their impact on the aminoacylation activity of AlaRS in vitro and/or in yeast (23, 24). Using a yeast complementation assay, all three mutations within the catalytic domain, including R329H, exhibited a loss of function in supporting yeast cell growth, suggesting these mutations abolished the aminoacylation activity of AlaRS (23, 24). The N71Y, R329H, and E778A mutations were also tested through an in vitro tRNA aminoacylation assay similar to what we perform here (23). While our findings on N71Y and E778A are consistent, the R329H mutation was found to severely reduce the tRNA charging activity of AlaRS (23). The discrepancy is hard to explain. However, we did note that the AlaRS constructs used in the previous study were fused to SMT3 protein at the N terminus to improve solubility, whereas our constructs do not have any N-terminal fusion or tag. There might be a slight chance for the fused SMT3 protein to selectively impact the R329H mutant. However, this cannot explain why R329H was found to be a loss-of-function mutation through the yeast complementation assay as well, where no fusion or tag was indicated for the construct (23). We should note that results from the yeast assay and from using the human proteins were found to be inconsistent for certain mutations in other CMT-linked aaRSs, including TyrRS (12, 37, 40), GlyRS (38, 41), and HisRS (7, 42), indicating yeast may not always be an accurate model for studying human mutations, at least for the tRNA synthetases.

Structurally speaking, despite the lack of a dimer interface, all mutations in the aminoacylation domain, whether or not affecting enzymatic activity, still induce a conformational opening of the catalytic core, as demonstrated by three independent biophysical measurements (i.e., HDX, SAXS, and switchSENSE). Remarkably, the open conformation of the AlaRS catalytic core enables interactions with Nrp1 (Fig. 6*A*), as does the open conformation of the GlyRS catalytic core (8, 9). We also tested HDAC6 and TRIM28 and found no aberrant interaction of AlaRS with these two candidates, suggesting a unique structural basis underlying the interaction with different partners and, at least for the Nrp1 interaction, dimer interface per se is not the key.

In contrast to the sticky mutation that causes Purkinje cell loss and ataxia by affecting the editing function of AlaRS, the E688G mutation in the editing domain does not lead to defective editing activity (Fig. 2*C*), ruling out the possibility that CMT2N is caused by toxicities linked to editing deficiency. Interestingly, the E688G mutation can also induce an open conformation, which however is different from that induced by mutations in the aminoacylation domain, as indicated by the HDX analysis (Fig. 4) and by its inability to make the aberrant Nrp1 interaction (Fig. 6*A*). This again highlights a specific structural change underlying the Nrp1 interaction and suggests that the direct molecular mechanism of E688G in causing CMT is likely to be different from that of the aminoacylation domain mutations. A lack of Nrp1 interaction was also observed for the GlyRS deltaETAQ mutant (43).

No functional and structural perturbations have been detected with the two C-Ala domain mutations at all (Figs. 2, 4, 5, and 6*A*). It is worth noting that one of the C-Ala mutations (E778A) is associated with incomplete penetrance and mild phenotypes (*SI Appendix*, Table S2) (23), suggesting insufficient genetic evidence for this mutation to be CMT causing. The other one (D893N), however, was identified in a large family and showed clear segregation with the neuropathy (*SI Appendix*, Table S2) (26). The clinical phenotypes associated with the D893N family seem unique. Compared with the typical distal motor and sensory neuropathy phenotypes associated with other CMT2N mutations, D893N patients exhibit distal hereditary motor neuropathy (dHMN) phenotypes only and have no sensory involvement (26). Moreover, among CMT-associated residues studied here, E778 and D893 are the least conserved ones across eukaryotes, bacteria, and archaea (Fig. 1*C*). In contrast, R329 is strictly conserved throughout evolution, correlating with its ultrastrong disease association. Whether and how mutations in the C-Ala domain are causatively linked to CMT remain to be further explored.

A prerequisite for a tRNA synthetase to interact with the extracellular region of Nrp1 would be its physical presence outside the cell. Our previous study showed GlyRS can be secreted from immortalized NSC-34 motor neuron cells and differentiated C2C12 myotubes (9). Interestingly, nine cytoplasmic aaRSs, including AlaRS, GlyRS, and the other three CMT-linked aaRSs were identified in the secretome of differentiating human myoblasts by mass spectrometry (44), suggesting secretion from muscle cells is a common feature of CMT-linked aaRSs. Similar to what we found for GlyRS (9), our preliminary study using transfected HEK293 cells suggests that CMT mutations do not affect the extracellular transportation of AlaRS (*SI Appendix*, Fig. S7 *A* and *B*). Although the impact of CMT mutations on AlaRS secretion should be further studied in disease-relevant cells and tissues, the result is consistent with the notion that the mutational impact on AlaRS-Nrp1 interaction we observed in transfected motor neuron cells and in patients’ lymphocytes is likely caused by changes in the ability rather than the accessibility of the mutant for the interaction.

Taken together, this structure–function study for AlaRS mutants confirmed that loss of function (enzymatic) is not the cause of CMT. CMT2N is also not linked to editing deficiency. We reveal that mutations in different aaRSs can lead to the same aberrant interaction, suggesting that Nrp1 is more broadly associated with CMT-associated members of the tRNA synthetase family. Moreover, the b1 domain of Nrp1 constitutes the consensus binding site for CMT-associated mutant AlaRS and GlyRS. Future study should investigate whether other aaRSs beyond GlyRS and AlaRS can also interact with the b1 domain of Nrp1 and how different aaRSs, with their apparently unique structures and sequences, can bind to the same receptor. Nevertheless, our result demonstrates the possibility that different tRNA synthetases can share the same gain-of-function disease-causing mechanism.

## Materials and Methods

Materials and general laboratory methods, protein expression and purification, thermal shift assay, in vitro transcription of tRNA, in vitro aminoacylation assay, patient lymphocyte immortalization, cell culture and growth, Northern blot, ATP-PPi exchange assay, crystallization and data collection, structure determination and refinement, small-angle X-ray scattering and ab initio shape reconstruction, switchSense analysis, HDX detected by MS, coimmunoprecipitation, domain mapping, and additional necessary information are available in *SI Appendix*, *SI Materials and Methods*.

## Supplementary Material

Supplementary File

## Data Availability

Coordinates and structure factors have been deposited in the Protein Data Bank for human AlaRS^N388^-WT and AlaRS^N388^-R329H under accession codes PDB 4XEM and PDB 4XEO, respectively.
