# Supplementary material for: CMT2N-causing aminoacylation domain mutants enable Nrp1 interaction with AlaRS
Source: Proc Natl Acad Sci U S A. 2021 Mar 22;118(13):e2012898118. doi: 10.1073/pnas.2012898118 (PMC8020758; doi:10.1073/pnas.2012898118)
Supplement: Supplementary File [file pnas.2012898118.sapp.pdf]

PNAS
www.pnas.org

Supplementary Information for

CMT2N-causing aminoacylation domain mutants enable Nrp1 interaction with AlaRS

Litao Sun[a,b,1], Na Wei [a,1], Bernhard Kuhle[a], David Blocquel[a], Scott Novick[c], Zaneta Matuszek[d], Huihao Zhou[a,e], Weiwei He[a,f], Jingjing Zhang[b], Thomas Weber[g], Rita Horvath[h], Philippe Latour[i], Tao Pan[d], Paul Schimmel[a,c,2], Patrick R Griffin[c], and Xiang-Lei Yang[a,2]

[a] Department of Molecular Medicine, The Scripps Research Institute, La Jolla, CA 92037;

[b] School of Public Health (Shenzhen), Sun Yat-sen University, 510006 Guangzhou, China;

[c] Department of Molecular Medicine, The Scripps Research Institute, Jupiter, FL 33458;

[d] Department of Biochemistry and Molecular Biology, University of Chicago, Chicago, IL 60637;

[e] School of Pharmaceutical Sciences, Sun Yat-sen University, 510006 Guangzhou, China;

[f] Shanghai Key Laboratory of New Drug Design, School of Pharmacy, East China University of Science and Technology, 200237 Shanghai, China;

[g] Dynamic Biosensors GmbH, 82152 Martinsried, Germany;

[h] Department of Clinical Neurosciences, University of Cambridge, Cambridge, CB2 0PY, United Kingdom;

[i] Biology and Pathology Department, Hospices Civils, Lyon, 68500, Bron, France

[1] These authors contributed equally to this work.

[2]Correspondence to Paul Schimmel, PhD (schimmel@scripps.edu) or Xiang-Lei Yang, PhD (xlyang@scripps.edu)

This PDF file includes:

Supplementary information text

Figures S1 to S7

Tables S1 to S2

SI References

Materials and methods

Expression and purification of AlaRS WT and CMT2N mutants

Human AlaRS and CMT2N mutants were cloned into pET-28a vector (Novagen). Constructs were transformed into BL21(DE3) cells. Cultures were grown overnight to saturation in LB medium containing 50 µg/ml Kanamycin. The overnight culture was diluted 1/100 in LB medium and grown at 37°C. Isopropyl ß-D-thiogalactopyranoside (IPTG) was added to a final concentration of 0.4 mM at OD_{600} of 0.5, and then the cells were grown at room temperature overnight. The cells were collected by centrifugation at 4,000rpm for 30min. The pellet was resuspended in lysis buffer (20 mM Tris-Cl, 300 mM NaCl, 5 mM Imidazole, 1 mM phenyl-methyl-sulphonyl-fluoride (PMSF), pH 8.0). The cells were disrupted by microfluidizer (M-110P) and the lysate was clarified by centrifugation at 28,000rpm for 30 min. AlaRS and all the recombinant CMT mutant proteins were purified using Ni-NTA beads (Qiagen), a HiTrap QHP column (GE Healthcare), and a HiLoad 16/60 Superdex 200 prep grade column (GE Healthcare). All purification steps were carried out at 4°C or on the ice.

Thermal shift assay

Thermal shifts assays were performed on a StepOnePlus 96 Real Time Cycler (Applied Biosystems). The dye from the Protein Thermal Shift™ Kit (Thermo Fisher Scientific) was used to monitor the thermal stability of protein by binding to the exposed hydrophobic regions. Solutions of 5ul of protein thermal shift buffer, 2.5ul of diluted thermal shift dye (8X) and 12.5ul of protein at 1 mg/ml were added to the wells of a 96-well Optical Reaction Plates (Applied Biosystems). The plates were sealed with optical sealing tape (Bio-Rad) and heated with the RT-PCR from 25 to 90℃ in increments of 0.5℃. Measurements were done in triplicate and analyzed with the corresponding Protein Thermal Shift™ Software. The florescence signal is monitored and plotted versus the temperature and the midpoint of the protein unfolding transition is defined as the T_m.

In vitro transcription of $tRNA^{Ala}$

DNA templates containing a T7 promoter and a $tRNA^{Ala}$ gene was synthesized by PCR of overlapping oligonucleotides(1). The transcription reaction was performed in 40 mM Tris-Cl pH 8.0, 25 mM NaCl, 20mM $MgCl_2$, 2 µg/ml pyrophosphatase, 0.1 mg/ml bovine serum albumin (BSA), 5mM DTT, 20 mM NTPs with T7 polymerase and the DNA template at 37 °C for 2 h. The tRNA transcript was purified by phenol-chloroform extraction and a HiTrap QHP column (GE Healthcare). The purified tRNA was annealed by heating up to 95 °C for 3 min and then slowly cooled down to room temperature with adding 1 mM $MgCl_2$ at 55°C.

In vitro aminoacylation assay

As described previously(1, 2)， the aminoacylation assays were performed at room temperature with 50 mM HEPES pH 7.5, 20 mM KCl, 5 mM $MgCl_2$, 4 mM ATP, 2mM DTT, 4 µg/ml pyrophosphatase, 20µM cold L-Alanine, 1.34 µM [^{3}H]-Alanine (1mCi/ml) as the experiment buffer. In vitro transcribed $tRNA^{Ala}$ was mixed with the assay buffer and the reaction was initiated by adding 200 nM AlaRS protein or 1µg/µL cell lysate into the mixture. The cell lysate was prepared in commercial lysis buffer (Thermo Fisher Scientific) with proteases inhibitors (Roche) and the concentration was measured by BCA method. At varying time intervals, 5 µL aliquots were applied to MultiScreen 96-well filter plate (0.45 um pore size Hydrophobic, low protein binding membrane (Millipore), which is pre-wetted with quench solution containing 0.5 mg/ml DNA and 100 mM EDTA in 300 mM NaOAc (pH 3.0). After the time points were collected, 100 µL 20% trichloroacetic acid (TCA) was added. The plate was washed four times by 5% TCA with 100 mM cold alanine and 95% ethanol once. After drying the plate, 70 µL of 100 mM NaOH was added, and then centrifuged into a 96-well flexible PET microplate (PerkinElmer) with 150 µL of Supermix scintillation cocktail (PerkinElmer). After mixing, the radioactivity in each well of the plate is counted in the 1450 LSC & Luminescence Counter (PerkinElmer).

Patient Lymphocyte Immortalization, Cell Culture, and Growth

For all the patients, 20 ml of peripheral blood were collected for generating immortalized lymphocyte. The samples were de-identified prior to use in our study. Within 48 hr of blood drawn, PBMC were isolated by density gradient centrifugation using Histopaque-1077 (Sigma-Aldrich), followed by removing red blood cells using RBC lysis buffer (Thermo Fisher Scientific) and cultured in RPMI-1640 media supplemented with 10% FBS and 1% penicillin streptomycin. Immortalization was accomplished by adding Epstein-Barr virus and cyclosporin (1:1000) to the resuspended PBMC followed by incubation at 37°C for one month. Immortalized lymphocytes were maintained in RPMI-1640 media with 10% FBS and 1% penicillin streptomycin. The cells were separated from the media by centrifugation at 500g for 5 min when collecting the lymphocytes.

Northern blot analysis

Oligonucleotide probes for Northern blots were ^{32}P-labeled with T4 PNK and purified by gel. The probe sequence is:

5’TGGAGAATGYGGGCGTCGATCCCRCTACCTCTYGCATGCTAAGCRAGCGCTCTAC CRCCTGAGCTAATTCCCC (R=A/G; Y=C/T) for nuclear encoded $tRNA^{Ala}$ (IGC).

Total RNA (2.5 μg) in 10 mM NaOAc/HOAc(pH 4.8) was mixed with the same volume of 8 M Urea, 0.1 M NaOAc/HOAc(pH 4.8), 0.05% Bromophenol blue, 0.05% Xylene cyanol. Total tRNA was deacylated in Tris-Cl (pH 9.0) at 37°C for 45 min. Samples were run on an 8% denaturing sequencing gel (8M Urea, 0.1M NaOAc/HOAc, pH 5.0) at 500V for 24 hr

at 4°C. RNA was transferred and fixed to Hybond-XL membrane (GE Healthcare) using a gel dryer at 80°C for 2 hr. The membrane was pre-hybridized twice at room temperature for 30 min in 20 mM sodium phosphate (pH 7.0), 300 mM NaCl, 1% sodium dodecyl sulphate (SDS). Hybridization of radiolabeled oligo (7 pmol) was performed in 20 mM sodium phosphate (pH 7.0), 300 mM NaCl, 1% SDS at 60°C for 16 hr. Membranes were washed three times in 20 mM sodium phosphate (pH 7.0), 300 mM NaCl, 0.1%SDS, 2 mM EDTA at 60°C for 20 min. The dried membranes were exposed to imaging plates and quantified by the phosphoimager (GE Healthcare).

ATP-PPi exchange assay

As described previously(2), the ATP/PPi-exchange assays were performed at room temperature with the assay solution, including 50 mM HEPES (pH 7.5), 20 mM KCl, 10 mM $MgCl_2$, 2mM DTT, 4 µg/ml pyrophosphatase. 1mM of Alanine, 2mM ATP, 1mM NaPPi and 0.001 µCi/µL [^{32}P] NaPPi were mixed with the assay solution, and the reaction was initiated by adding 200 nM AlaRS or R329H mutant proteins into the mixture. At varying time intervals, 15 µL aliquots were applied to MultiScreen 96-well filter plate (0.45 um pore size hydrophobic, low protein binding membrane, (Millipore)), which is pre-wetted with quench solution containing 1M HCl, 200 mM sodium pyrophosphate, 4% charcoal. The plate was washed by wash solution (1 M HCl, 200 mM sodium pyrophosphate) four times and then dried. The wells of the plate were punched into small scintillation vials with 3 mL scintillation solution and incubated for 30 min. The radioactivity in each vial was counted in the scintillation counter.

Crystallization and data collection

Crystallization experiments were performed immediately after protein purification. Before setting up crystallization, 2 mM Ala-AMP analog (5’-O-[N-(L-Alanyl)-sulfamoyl]adenosine) AlaSA (Integrated DNA Technologies) was added into hAlaRS-N^{455}-R329H protein solution, and incubated on ice for one hr. The proteins were crystallized with sitting-drop vapor diffusion method. Each drop contained 100 nL of 30mg/ml protein and 100 nL reservoir solution, and equilibrated against 70µL reservoir solution. hAlaRS-N^{388}-R329H crystals (the fragment was from hAlaRS-N^{455}-R329H protein) were grown with the reservoir solution (30% PEG3,350, 100 mM HEPS pH 7.5) at 20 °C. The purified wild type hAlaRS-N^{455} was treated with 1/2000 (m/m)

trypsin at 4 °C overnight. After tryspin digestion, the largest fragment(hAlaRS-N^{388}), which runs about 40 kDa on SDS-PAGE, was further purified by mono Q ion-exchange chromatography. hAlaRS-N^{388} was concentrated to 30 mg/ml and then incubated with 2 mM AlaSA on ice for one hr. hAlaRS-N^{388} was crystallized using reservoir solution (0.2M $MgCl_2$, 20% PEG3350) at 20 °C. Diffraction data were collected at 100 K on beamline 11-1 of Stanford Synchrotron Radiation Lightsource (SSRL) and processed with program HKL2000.

Structure determination and refinement

Crystal structures of hAlaRS-N^{388}(wild type or R329H) were solved with molecular replacement method by program MOLREP and *E. coli* AlaRS catalytic domain structure (PDB code: 3HXU) was used as the searching template. Structure rebuilding and refinement were performed in COOT and REFMAC5. The wild type hAlaRS-N^{388} structure was finally refined to 1.28 Å resolution with R_{work}=16.1% and R_{free}=17.9%, and the R329H mutant hAlaRS-N^{388} structure was refined to 1.38 Å with R_{work}=18.7% and R_{free}=21.5%. The model quality was validated using MolProbity. Data collection and structure refinement statistics are summarized in Supplementary Table S1.

Small-angle X-ray scattering (SAXS) and *ab initio* shape reconstruction

As described previously(3), the SAXS measurements were performed at the SSRL on beamline BL4-2 at a working energy of 12.5 KeV and buffer background runs were subtracted from sample runs. The forward scattering intensity was calibrated using bovine serum albumin as a reference. The sample-to-detector distance of the X-rays was 2.847 m, leading to scattering vectors q ranging from 0.028 to 4.525 nm^{-1}. The exposure time was optimized to reduce radiation damage. Data reduction was performed using the established procedure available at the beamline. The radius of gyration (R_g) and forward intensity at zero angle $I(0)$, were determined with the program PRIMUS (4) according to the Guinier approximation at low values.

The forward scattering intensity was calibrated using bovine serum albumin as reference. The R_g and pairwise distance distribution function, P(r), were calculated with the program GNOM. The maximum dimension (D_{max}) value was adjusted that the R_g value obtained from GNOM agreed with that obtained from the Guinier analysis.

Three-dimensional bead models were built by fitting the scattering data with the program DAMMIF(5). Thirty independent models were generated with DAMMIF assuming P2 symmetry. The models resulting from independent runs were superimposed using the DAMAVER suite(6). This yielded an initial alignment of structures based on their axes of inertia, followed by minimization of the normalized spatial discrepancy, which is zero for

identical objects and larger than one for systematically different objects. The aligned structures were averaged, giving an effective occupancy to each voxel in the model, and filtered at half-maximal occupancy to produce models of the appropriate volume that were used for all subsequent analyses.

SwitchSense analysis

Kinetic measurements were performed using a DRX series instrument (Dynamic Biosensors) with a MPC-48-2-Y1 chip (Dynamic Biosensors) as described previously(3). All AlaRS proteins were dialyzed by T40 buffer (10 mM Tris–HCl pH 7.4, 40 mM NaCl and 0.05% tween20). A reporter fluorophore has been attached to the DNA's distal end, and sizing experiments were performed with an association time of 20 min at a flow rate of 50 μL/min. We verified that 150nM AlaRS was observed no interaction with scrambled equivalent RNAs (SCRM ×1 to ×4). For sizing, the time-resolved fluorescence intensity signal corresponding to the upward motion of the DNA nanolevers was evaluated as described (7) using switchANALYSIS software (Dynamic Biosensors GmbH, Planegg, Germany). The size analysis allowed estimation of an apparent size value for all the tested proteins.

Hydrogen-deuterium exchange (HDX) detected by mass spectrometry (MS)

Differential HDX-MS experiments were conducted as previously described with a few modifications (8, 9).

Peptide identification: Peptides were identified using tandem MS (MS/MS) with an Orbitrap mass spectrometer (Q Exactive, ThermoFisher). Product ion spectra were acquired in data-dependent mode with the top five most abundant ions selected for the product ion analysis per scan event. The MS/MS data files were submitted to Mascot (Matrix Science) for peptide identification. Peptides included in the HDX analysis peptide set had a MASCOT score greater than 20 and the MS/MS spectra were verified by manual inspection. The MASCOT search was repeated against a decoy (reverse) sequence and ambiguous identifications were ruled out and not included in the HDX peptide set.

HDX-MS analysis: 5 μL of 10 μM protein was diluted into 20 μL D_2O buffer (20 mM Tris-HCl, pH 7.4; 150 mM NaCl; 2 mM DTT) and incubated for various time points (0, 10, 60, 300, and 900 s) at 4°C. The deuterium exchange was then slowed by mixing with 25 μL of cold (4°C) 3 M urea and 1% trifluoroacetic acid. Quenched samples were immediately injected into the HDX platform. Upon injection, samples were passed through an immobilized pepsin column (2mm × 2cm) at 200 μL/min and the digested peptides were captured on a 2mm × 1cm C_8 trap column

(Agilent) and desalted. Peptides were separated across a 2.1mm × 5cm C_{18} column (1.9 µL Hypersil Gold, ThermoFisher) with a linear gradient of 4% - 40% CH_3CN and 0.3% formic acid, over 5 min. Sample handling, protein digestion and peptide separation were conducted at 4°C. Mass spectrometric data were acquired using an Orbitrap mass spectrometer. HDX analyses were performed in triplicate, with single preparations of each protein. The intensity weighted mean m/z centroid value of each peptide envelope was calculated and subsequently converted into a percentage of deuterium incorporation. This is accomplished determining the observed averages of the undeuterated and fully deuterated spectra and using the conventional formula described elsewhere(10). Statistical significance for the differential HDX data is determined by an unpaired t-test for each time point, a procedure that is integrated into the HDX Workbench software(11). Corrections for back-exchange were made on the basis of an estimated 70% deuterium recovery, and accounting for the known 80% deuterium content of the deuterium exchange buffer.

Data Rendering: The HDX data from all overlapping peptides were consolidated to individual amino acid values using a residue averaging approach. Briefly, for each residue, the deuterium incorporation values and peptide lengths from all overlapping peptides were assembled. A weighting function was applied in which shorter peptides were weighted more heavily and longer peptides were weighted less. Each of the weighted deuterium incorporation values were then averaged to produce a single value for each amino acid. The initial two residues of each peptide, as well as prolines, were omitted from the calculations. This approach is similar to that previously described(12).

HDX analyses were performed in triplicate, with single preparations of each purified protein. Statistical significance for the differential HDX data is determined by t test for each time point, and is integrated into the HDX Workbench software(11).

Sample processing protocol: 5 µL of 10 µM AlaRS (WT or CMT mutant) was diluted into 20µL of protein storage buffer, quenched by mixing with 25 µL of 3M urea and 1% trifluoroacetic acid. Each sample was passed through an immobilized pepsin column, and the digested peptides were captured on a C_8 trap column and desalted. Peptides were separated across a C_{18} column with a linear gradient of 4-40% CH3CN and 0.3% formic acid, with a gradient of 5 min for HDX and one hr for MSMS. Mass spectrometric data were acquired using a Q Exactive mass spectrometer.

Co-immunoprecipitation

NSC-34 cells transfected with V5-tagged human AlaRS constructs and patient-derived and control lymphocytes were lysed with Pierce IP Lysis Buffer (Thermo Scientific) for 30 min. The cell lysate was separated by centrifugation at 15,600 g for 7 min. Separately, rProtein G agarose beads (Invitrogen) were incubated with a rabbit monoclonal antibody to Neuropilin 1 (Abcam 81321) or normal IgG rabbit antibody (Cell Signaling Technologies) for 30 min. The cell lysate is incubated with washed beads for 3 hr at 4°C. After incubation, the beads are washed 3 times with TNT wash buffer (20 mM Tris-HCl, 150 mM NaCl, 0.1% Triton-X 100, 1mM DTT, 1x Roche protease inhibitor), boiled in SDS sample loading buffer, and then the supernatant containing bound proteins were separated from the beads by centrifugation.

Domain mapping of the AlaRS^{CMT}-Nrp1 interaction

The constructs of Nrp1 used for pull-down experiments include the intact extracellular domain (residues Arg23–Asp840), b1b2c domains (residues Phe273-Asp840), a1a2 domains (residues Arg23-Asp272), b1b2 domains (residues Phe273-Phe643), c domain (residues Thr589-Asp840), b1 domain (residues Phe273-Asp428) and the b2 domain (residues Lys425-Phe643), cloned with a C-terminal human IgG Fc-tag into the pcDNA6.0/V5-His-A vector (Life Technologies) as described previously(13). Human AlaRS and Nrp1 constructs were each transfected into NSC-34 cells in a 6-well plate 48 hr after transfection, cells were lysed with Pierce IP Lysis Buffer for 30 min on ice. The cell lysate was then centrifuged at 15,600 g for 10 min, and the supernatant containing Nrp1 protein was incubated with 30 µl Protein A resin (Invitrogen). The Nrp1-bound resins were divided equally into two 1.5 ml Eppendorf tubes and incubated with the lysate from NSC-34 cells transfected with either WT or R239H AlaRS (lysed in Pierce IP Lysis Buffer) for 3 hr. Resins were then washed three times with TNT wash buffer. The bound proteins were eluted with 30 µl of SDS sample loading buffer and subjected to western blotting analysis using mouse anti-AlaRS (Santa Cruz Biotechnology, sc-165990; 1:1,000) and goat anti-human IgG antibodies (Abcam, ab98624 ; 1:10,000) to detect AlaRS and the Nrp1 variants, respectively.

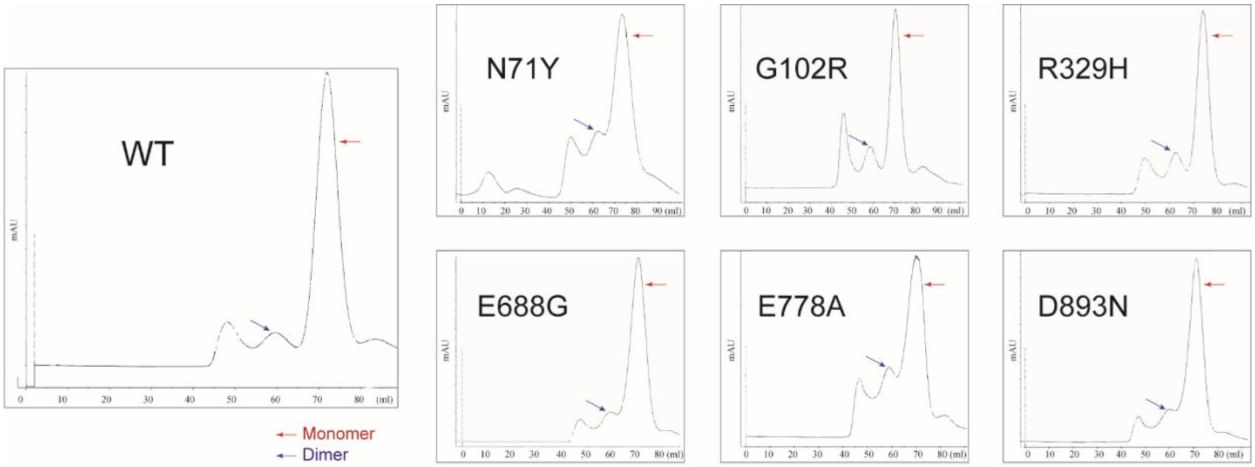

Figure S1. CMT mutations appear to not affect AlaRS oligomerization.
Purified recombinant AlaRS proteins were analyzed by gel filtration chromatography in the buffer containing 20 mM HEPES (pH 8.0), 250 mM NaCl, and 2 mM DTT.

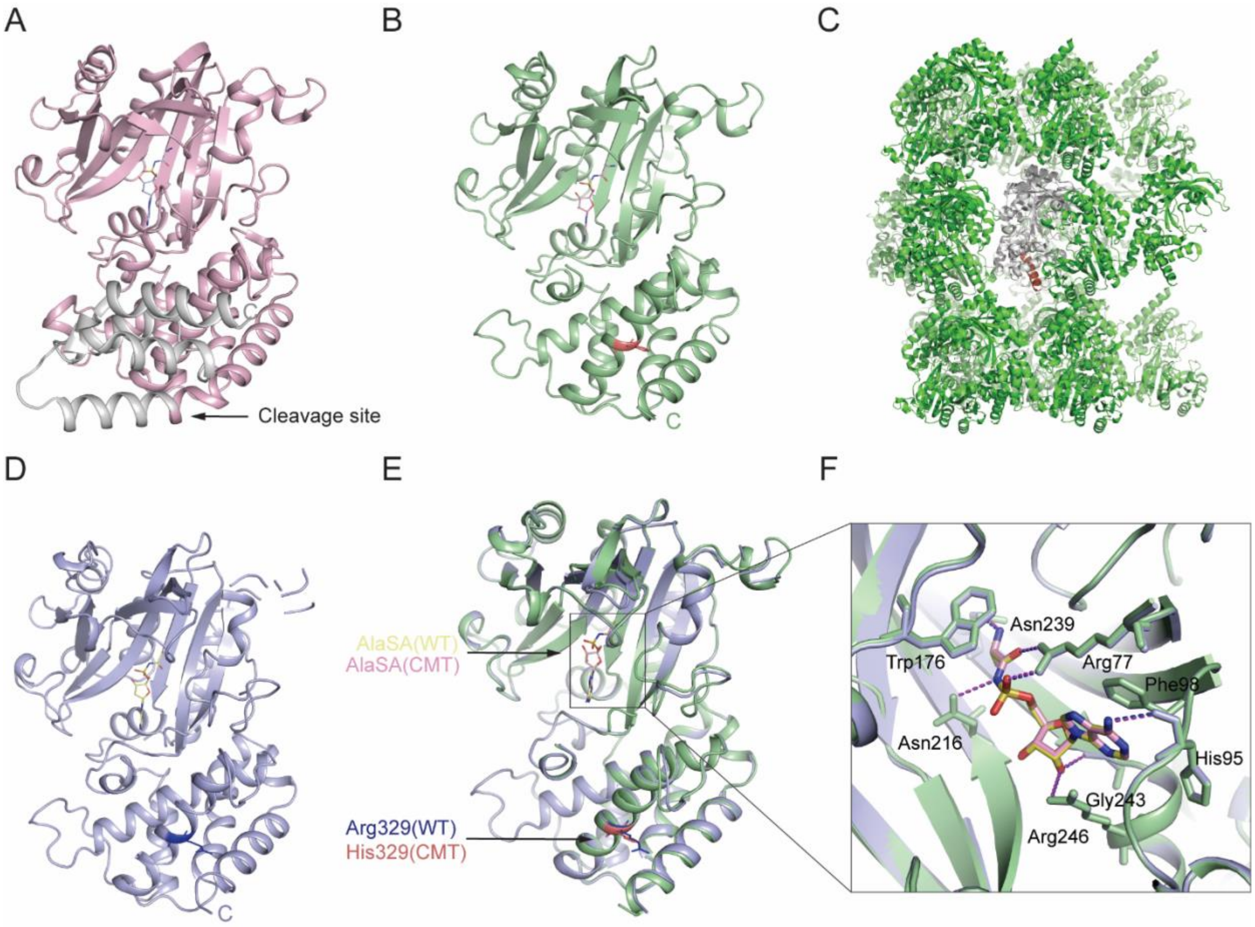

Figure S2. Crystal structures of human WT and R329H AlaRS.

(A) Co-crystal structure of human AlaRS^{N455} and AlaSA (PDB: 5KNN). AlaRS^{455} is shown in cartoon with amino acids T4-R388 in pink color and G389-Q453 in grey color, while AlaSA was shown in light blue for carbon atoms. **(B)** Co-crystal structure of human AlaRS^{N388}-R329H and AlaSA solved at 1.38 Å (PDB: 4XEO). AlaRS^{N388}-R329H is shown in cartoon with light green color, while AlaSA (purple for carbon atoms) and His329 (salmon) was shown in stick. **(C)** One of AlaRS^{N388}-R329H molecules was shown in grey with the last α helix in the C-terminal in red color, while the other molecules are labelled in green in the crystal lattice. **(D)** Co-crystal structure of human AlaRS^{N388} and AlaSA solved at 1.28 Å (PDB: 4XEM). AlaRS^{N388} is shown in cartoon with light blue color, while AlaSA (yellow for carbon atoms) and residue Arg329 (dark blue) was shown in stick. **(E)** Superimposition of AlaRS^{N388} (in light blue; PDB 4XEM) with AlaRS^{N388}-R329H (in light green; PDB 4XEO). **(F)** A zoom-in view showing the AlaSA-bound catalytic pocket is essentially identical between WT- and R329H-AlaRS^{N388}.

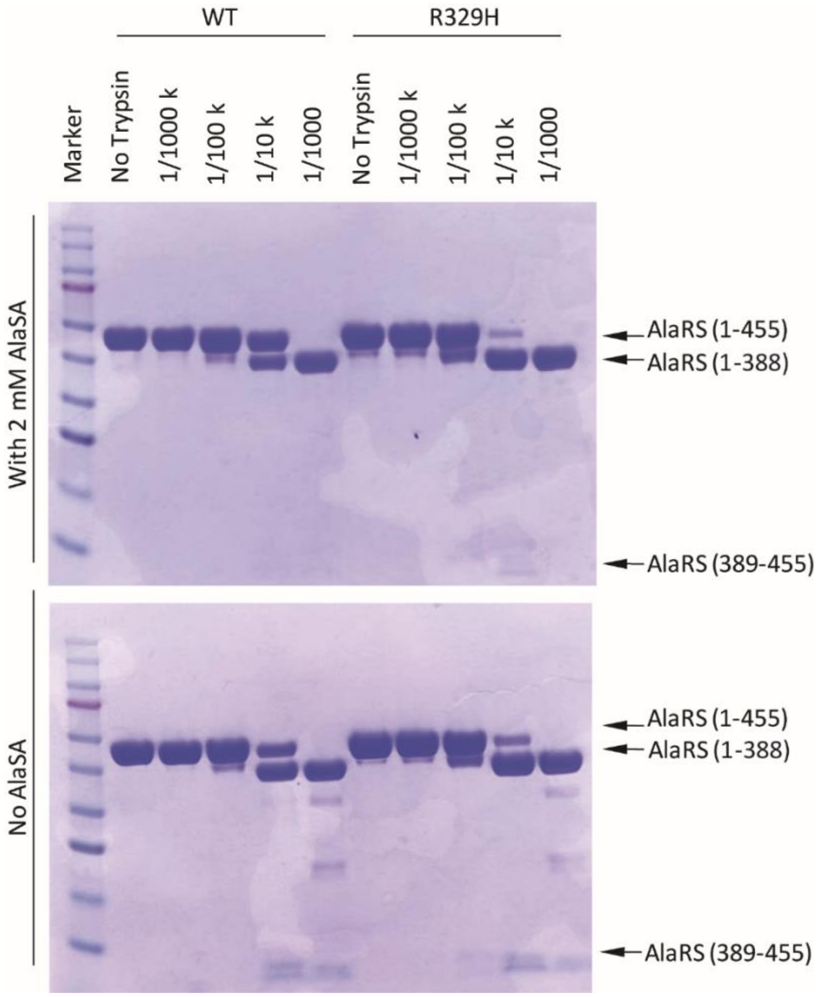

Figure S3. Limited Proteolysis analysis of WT and R329H AlaRS.

The AlaRS proteins (in the presence and absence of AlaSA) were incubated with trypsin in different concentrations for 20 min before the reactions were quenched, and the products were separated by SDS-PAGE.

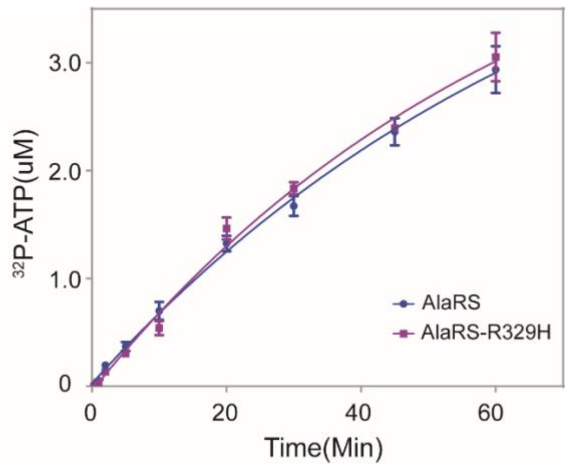

Figure S4. ATP-pyrophosphate exchange analysis of WT and R329H AlaRS.

The reaction was performed with purified recombinant AlaRS proteins. Data are presented as mean ± SD (n=2).

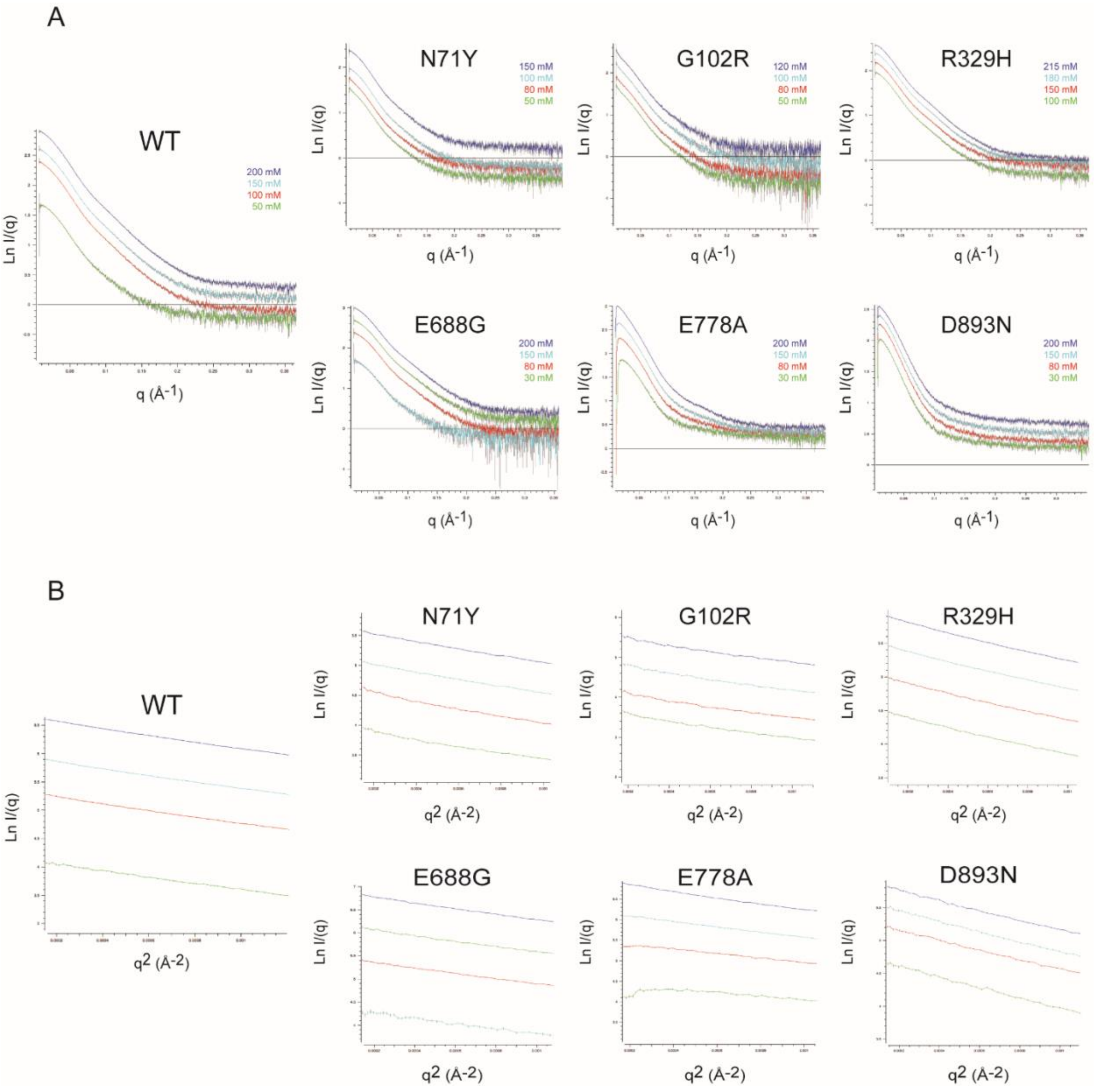

Figure S5: Small-angle X-ray scattering raw spectra and Guinier analysis.

(A) SAXS recorded for q values up to 0.35 Å $^{-1}$ for WT and CMT2N AlaRS proteins. The curves obtained at different concentrations are represented without correction for concentration. **(B)** Representation of the corresponding Guinier plot at each of the concentrations tested.

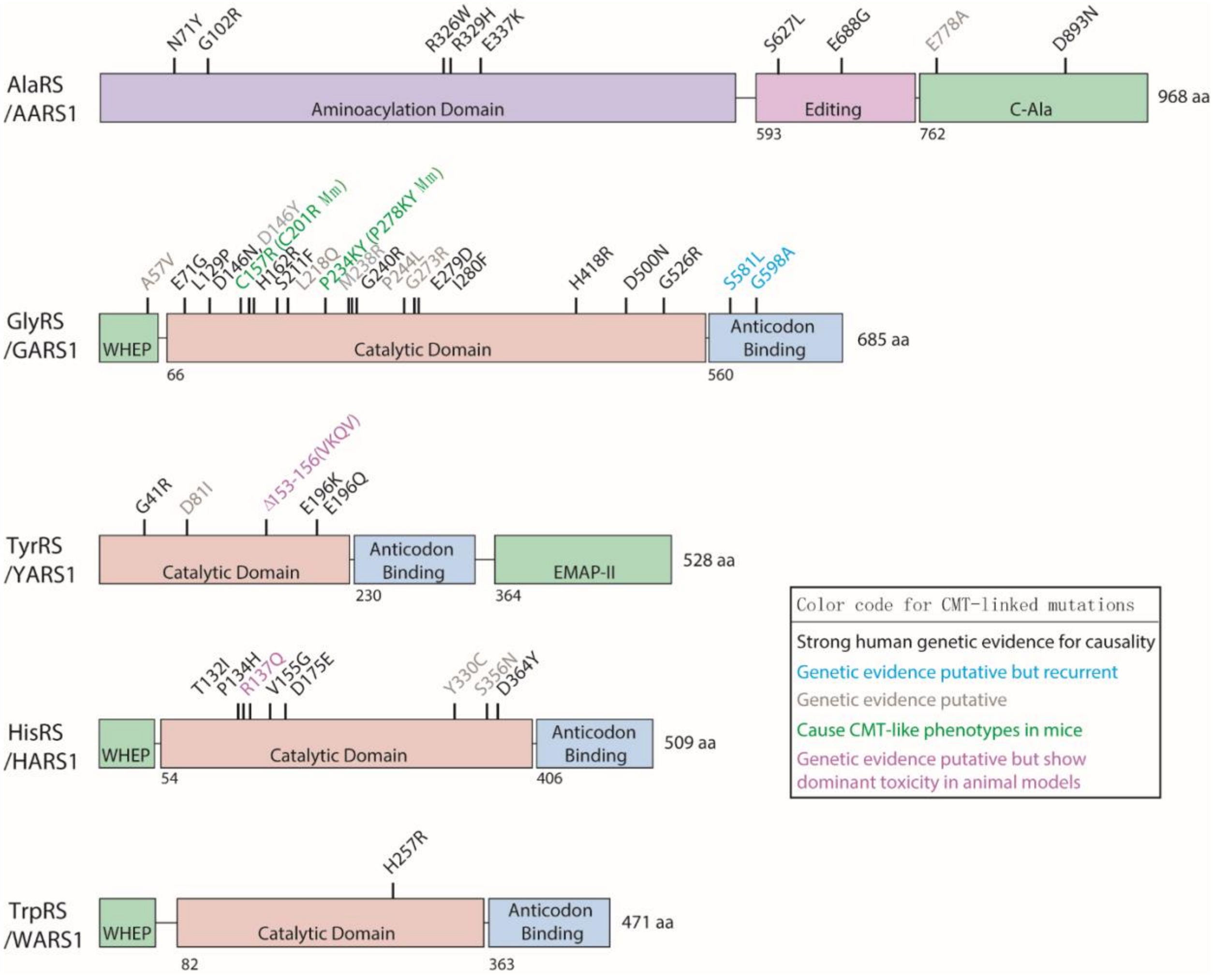

Figure S6. Distribution of CMT-linked mutations in aaRSs.

For GlyRS, the mutations are numbered according to the cytosolic form.

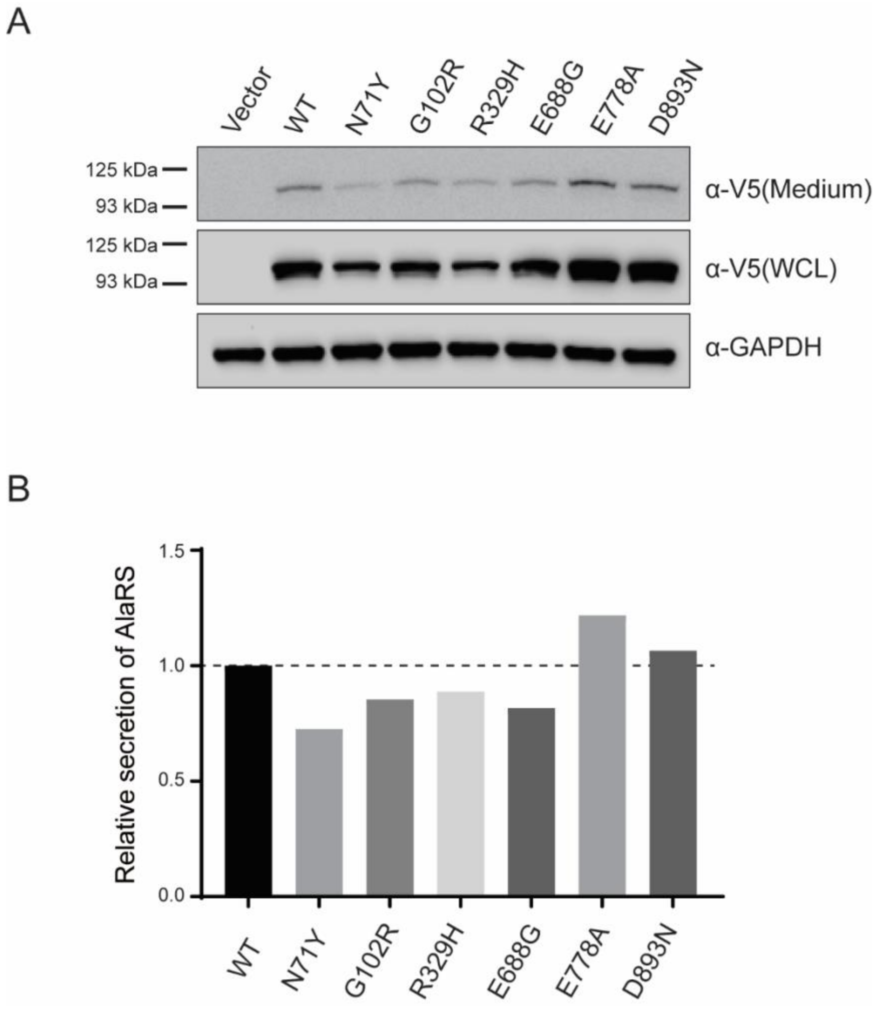

Figure S7. The extracellular transportation of CMT mutations in AlaRS.

(A) The western blot showing CMT-mutations do not affect the secretion of AlaRS. **(B)** Relative amounts of extracellular transported AlaRS is quantified based on the amount of secreted AlaRS relative to the amount of AlaRS in the whole cell lysate (WCL) .

Supplementary Table S1. Data collection and refinement statistics.

Crystal	AlaRS^{N388}	AlaRS ^{N388}-R329H
**Data collection**	4XEM	4XEO
Resolution (Å)	50.0-1.28(1.30-1.28)[a]	50.0-1.38(1.40-1.38)
Wavelength (Å)	1.0000	0.9795
Space group	*C*2	$P2_1$
Unit cell a, b, c (Å)	*a*=108.12, *b*=67.13, *c*=75.11	*a*=64.85, *b*=121.17, *c*=68.01
Unit cell α, β, γ (°)	*α*=*γ*=90, *β*=127.42	*α*=*γ*=90, *β*=118.47
Unique reflections	109596(5498)	185858(7807)
Redundancy	3.5(3.4)	3.6(2.4)
Completeness (%)	99.8(99.9)	98.3(82.9)
Average I/σ(I)	35.5(2.9)	29.0(2.6)
R_{merge}[b] (%)	4.0(48.3)	3.8(40.3)
**Refinement**		
Resolution (Å)	37.33-1.28(1.31-1.28)	33.96-1.38(1.42-1.38)
Reflections for refinement/test	104085/5511	176334/9339
R_{work}[c]/R_{free}[d] (%)	16.1/17.9(25.5/27.7)	18.7/21.5(26.8/31.1)
RMSD bond (Å)	0.006	0.008
RMSD angle (°)	1.08	1.11
Mean B factor (Å^2)	18.0	20.4
Non-hydrogen protein atoms	3057	6264
Water oxygen atoms	489	810
Other Non-hydrogen atoms	48	102
Molprobity Ramachandran plot (%)		
Most favored regions	98.2	98.3
Additional allowed regions	100.0	100.0

[a]Values in parentheses are for the highest resolution shell.

[b]$R_{merge} = \Sigma_h \Sigma_l \mid I(h)_l - \langle I(h)\rangle \mid / \Sigma_h \Sigma_l I(h)_l$, where $I(h)_l$ is the *l*th observation of the reflection h and $\langle I(h)\rangle$ is the weighted average intensity for all observations *l* of reflection h.

[c]$R_{work}=\sum_h \mid \mid F_{obs}(h) \mid - \mid F_{cal}(h) \mid \mid / \sum_h \mid F_{obs}(h) \mid$, where $F_{obs}(h)$ and $F_{cal}(h)$ are the observed and calculated structure factors for reflection h respectively.

[d]R_{free} was calculated as R_{work} using the 5% of reflections which were selected randomly and omitted from refinement.

Supplementary Table S2. Clinical information of CMT2N patients.

Gene	Affected domain	Mutation	Penetrance	Genetic evidence and clincial features	Onset age	References
AARS	Catalytic	N71Y	Complete	Strong - muation segregates with 7 patients in a 3-generation Taiwanese family; distal weakness and wasting, mild sensory loss.	11-30 y	(14)
		G102R	Complete	Strong - mutation segregates with 5 patients in a 3-generation Belgian family; mild axonal neuropathy; 4 of 5 patients had a myelopathy.	22-46 y	(15)
		R329H	Complete	Very strong - mutation segregates with disease in 8 unrelated, multiple generations CMT families from 4 countries (France, Australia, UK, Ireland); motor (predominant) and sensory neuropathy, some patients have demyelination (intermediate NCV).	6-54 y	(16-18)
	Editing	E688G	Unclear	Strong - mutation segregates with 3 patients in a 3-generation Irish family; early onset motor (predominant) and sensory neuropathy with intermediate NCV.	<1-10 y	(16)
	C-Ala	E778A	Incomplete	Putative - sensorimotor axonal neuropathy in an Australian proband, while only rippling muscles and cramps were present in three affected relatives.	n/a	(18)
		D893N	Complete	Strong - mutation segregates with 4 patients in a 3-generation Chinese family; distal motor neuropathy (dHMN) with normal NCV and without sensory involvement; fatty infiltration of the gastrocnemius and vastus lateralis muscles.	11-55y	(19)

Reference

1. M. H. Nawaz, E. Merriman, X. L. Yang, P. Schimmel, p23H implicated as cis/trans regulator of AlaXp-directed editing for mammalian cell homeostasis. *Proc Natl Acad Sci U S A* **108**, 2723-2728 (2011).
2. K. Beebe, W. Waas, Z. Druzina, M. Guo, P. Schimmel, A universal plate format for increased throughput of assays that monitor multiple aminoacyl transfer RNA synthetase activities. *Anal Biochem* **368**, 111-121 (2007).
3. D. Blocquel *et al.*, CMT disease severity correlates with mutation-induced open conformation of histidyl-tRNA synthetase, not aminoacylation loss, in patient cells. *Proc Natl Acad Sci U S A* **116**, 19440-19448 (2019).
4. P. V. Konarev, V. V. Volkov, A. V. Sokolova, M. H. J. Koch, D. I. Svergun, PRIMUS: a Windows PC-based system for small-angle scattering data analysis. *J Appl Crystallogr* **36**, 1277-1282 (2003).
5. D. Franke, D. I. Svergun, DAMMIF, a program for rapid ab-initio shape determination in small-angle scattering. *J Appl Crystallogr* **42**, 342-346 (2009).
6. V. V. Volkov, D. I. Svergun, Uniqueness of ab initio shape determination in small-angle scattering. *J Appl Crystallogr* **36**, 860-864 (2003).
7. A. Langer *et al.*, Protein analysis by time-resolved measurements with an electro-switchable DNA chip. *Nat Commun* **4**, 2099 (2013).
8. W. He *et al.*, Dispersed disease-causing neomorphic mutations on a single protein promote the same localized conformational opening. *Proc Natl Acad Sci U S A* **108**, 12307-12312 (2011).
9. M. J. Chalmers *et al.*, Probing protein ligand interactions by automated hydrogen/deuterium exchange mass spectrometry. *Anal Chem* **78**, 1005-1014 (2006).
10. Z. Zhang, D. L. Smith, Determination of amide hydrogen exchange by mass spectrometry: a new tool for protein structure elucidation. *Protein Sci* **2**, 522-531 (1993).
11. B. D. Pascal *et al.*, HDX workbench: software for the analysis of H/D exchange MS data. *J Am Soc Mass Spectrom* **23**, 1512-1521 (2012).
12. T. R. Keppel, D. D. Weis, Mapping residual structure in intrinsically disordered proteins at residue resolution using millisecond hydrogen/deuterium exchange and residue averaging. *J Am Soc Mass Spectrom* **26**, 547-554 (2015).
13. W. He *et al.*, CMT2D neuropathy is linked to the neomorphic binding activity of glycyl-tRNA synthetase. *Nature* **526**, 710-714 (2015).
14. K. P. Lin *et al.*, The mutational spectrum in a cohort of Charcot-Marie-Tooth disease type 2 among the Han Chinese in Taiwan. *PLoS One* **6**, e29393 (2011).
15. W. W. Motley *et al.*, A novel AARS mutation in a family with dominant myeloneuropathy. *Neurology* **84**, 2040-2047 (2015).
16. B. Bansagi *et al.*, Genotype/phenotype correlations in AARS-related neuropathy in a cohort of patients from the United Kingdom and Ireland. *J Neurol* **262**, 1899-1908 (2015).

17. P. Latour *et al.*, A major determinant for binding and aminoacylation of tRNA(Ala) in cytoplasmic Alanyl-tRNA synthetase is mutated in dominant axonal Charcot-Marie-Tooth disease. *Am J Hum Genet* **86**, 77-82 (2010).
18. H. M. McLaughlin *et al.*, A recurrent loss-of-function alanyl-tRNA synthetase (AARS) mutation in patients with Charcot-Marie-Tooth disease type 2N (CMT2N). *Hum Mutat* **33**, 244-253 (2012).
19. Z. Zhao *et al.*, Alanyl-tRNA synthetase mutation in a family with dominant distal hereditary motor neuropathy. *Neurology* **78**, 1644-1649 (2012).
